# Second Harmonic Modulation for Ultrasonic Signals Based on the Design of the Phononic Crystal Filter

**DOI:** 10.3390/s23229227

**Published:** 2023-11-16

**Authors:** Yue Zhu, Youxuan Zhao, Peng Cao

**Affiliations:** 1 School of Aeronautics, Northwestern Polytechnical University, Xi’an 710072, China; zhuyuezy@mail.nwpu.edu.cn; 2 College of Aerospace Engineering, Chongqing University, Chongqing 400044, China; 3 Faculty of Architecture, Civil and Transportation Engineering, Beijing University of Technology, Beijing 100124, China; caopeng518888@126.com

**Keywords:** nonlinear ultrasonic, ultrasonic filter, phononic crystal, bandgaps properties, numerical simulation

## Abstract

Nonlinear ultrasonic non-destructive testing (NDT) is a widely used method for detecting micro-damages in various materials and structures due to its high sensitivity and directional capability. However, the extraction and modulation of extremely weak nonlinear ultrasonic signals is quite a challenge in practical applications. Therefore, this paper focuses on the second harmonic modulation signal method in nonlinear ultrasonic NDT and proposes the design of the phononic crystal filter (PC filter) to achieve this filtering function. Through finite element simulations, it is demonstrated that the filtering frequency of the filter is influenced by the structural configuration, material wave speed, and geometric characteristics. Then, the design method for cubic PC filters is established. Furthermore, a time-domain finite element method is introduced to verify the filtering ability of the filter and further validate the rationality of this design approach.

## 1. Introduction

Periodic (or topological lattice) phononic crystals (PCs) and acoustic metamaterials wield a profound influence on wave propagation. Metamaterials are structures endowed with artificially crafted properties that natural materials cannot replicate [1]. These unique attributes encompass sound blocking [2], comprehensive sound absorption [3,4], acoustic lensing [5,6] and sound invisibility [7]. Phononic crystals, constituting a vital subfield within metamaterials, are chiefly dedicated to controlling the behavior of elastic waves [8]. Recent advancements have led to the creation of innovative structures customized to cater to diverse industry needs. For instance, Quinteros et al. [9] devised a methodology for optimizing the topology of sandwich panels by employing cellular truss cores, thereby enhancing vibration absorption in honeycomb structures commonly used in extensive transportation vehicles such as aircraft. Furthermore, practical applications have been explored in compartments [10] and UAV formation control [11]. As research progresses, the pursuit of broad-frequency characteristics has yielded remarkable metamaterials. Inspired by biomaterials, Zhang et al. [12] engineered a straightforward multilayered model with wide bandgap features.

The bandgap characteristics (frequency band properties) of PCs hold significant importance within this field due to their ability to impede the transmission of specific frequency signals through these crystals [13]. Among them, the bandgap property is a fundamental feature, rendering certain frequency signals incapable of passing through PCs. It is regarded as a vital attribute of PCs. Consequently, research on how to control and harness these bandgap properties for the purpose of filtering specific signals has become a central focus in the field of PCs [14]. For example, Nguyen et al. [15] designed the double-layer membrane-type metamaterials for low-frequency sound reduction, which possesses broadband properties at 0.32~2.5 kHz. This study is concentrated on the bandgap characteristics under the Bragg mechanism, aiming to explore the full utilization of this mechanism. As the understanding of the bandgap properties of PCs has deepened, researchers have begun to contemplate how these periodic structures can be employed to achieve physical control over signals [16,17]. At present, periodic metamaterials have been widely studied in the field of non-destructive testing (NDT) and structural health monitoring (SHM) [18].

**Figure 1:**
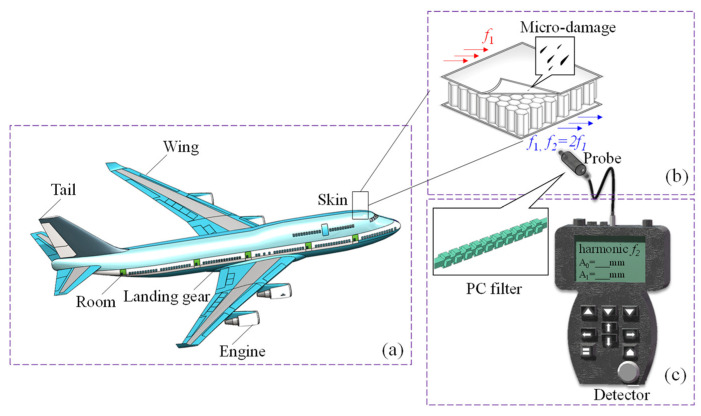
The engineering application of PC filter. (**a**) Normal part with high micro-damage. (**b**) The scheme of nonlinear ultrasonic nondestructive testing of second harmonic method. (**c**) The PC filter reserves the second harmonic signal which includes the characteristic of damage.

Non-Destructive Testing (NDT) encompasses various methods, including visual inspection (VI), acoustic emission (AE), and ultrasonic testing (UT) [19]. Among these, Nonlinear Ultrasonic Testing (NUT) plays a significant role in early damage detection [20,21]. As representative nonlinear ultrasonic techniques, the high harmonic method [22,23,24] and non-collinear wave mixing method [25,26,27] are commonly employed. Quantitative damage assessment [28,29], composite materials testing [30,31], and damage localization [32] are hot issues in nonlinear ultrasonic NDT. However, nonlinear signals often come with unavoidable nonlinear interference introduced by external factors. The complexity of these signals poses significant challenges to traditional signal processing methods, such as the Fourier transform. Wang et al. [33] demonstrated the bandgap properties of Lamb waves in two-dimensional PC plates by using laser-ultrasonic non-destructive testing methods. Therefore, the design of PCs is crucial in advancing the field of nonlinear ultrasonic non-destructive testing. In recent years, some researchers have begun to explore the application of metamaterials in filter design [34,35]. Compared with digital filters, the use of physical filters allows for real-time adjustments in filter placement based on specific measurement needs, all without introducing additional noise. As depicted in Figure 1, materials commonly employed in the aerospace industry include aluminum alloys and composite materials, which are prone to developing microscopic cracks during operation [36]. Even small cracks can pose a serious threat to flight safety, particularly in extreme atmospheric conditions [37]. Consequently, regular structural health monitoring has become exceptionally important. We believe that the incorporation of physical filters, i.e., the phononic crystal filter (PC filter), into handheld signal detection devices will significantly enhance monitoring efficiency and accuracy.

The field of NUT pays great attention to the high-frequency bandgap characteristics of PCs, but the research in this area remains limited at present. Lee et al. [38] proposed hierarchical PCs to regulate the ultrasonic signal of 20 kHz to 10 MHz. Iglesias Martínez et al. [39] presented a three-dimensional phononic crystal with a cubic symmetry operating in the frequency band from 0.6 MHz to 7.5 MHz, which can be fabricated by two-photon lithography at the microscale. Elizabeth J. et al. [40] Proposed a PC’s ultrasonic filter for nonlinear ultrasonic testing, which exhibited distinct ultrasonic frequency bandgaps characteristics. The model is based on three-dimensional PCs and considers the effect of actual processing on the bandgap characteristics. Although remarkable progress has been made, the practical application of these previous studies is still challenging. In the latest research, Liu et al. [41] proposed to use the Deep Learning method to solve the band gap design problem of phononic plate metamaterial, and the design band gap frequency is about 3 kHz, which is a kind of low-frequency design amplification. However, these studies mainly focused on the PCs’ designation or low-frequency range, as shown in Table 1. At present, the research and summary of the design method of band gap in ultrasonic frequency is rarely reported. Therefore, aiming at an existing phononic crystal with simple cubic configuration, we propose a design method to obtain the target bandgap frequency of the structure, which could be applied to the pre-design of the signal filter for ultrasonic nonlinear detection. In order to verify the effectiveness of the design method, the relevant design parameters are analyzed, and the influence of machining error on the filtering ability is considered.

The structure of this paper is organized as follows: The filter design method based on the target bandgap frequency’s empirical formula is presented in Section 2. Next, simulation methods for verifying bandgap characteristics and filtering capabilities are introduced in Section 3. Then, Section 4 conducts the detailed parameter study of the filter’s intrinsic mechanism, including the influence of geometry, materials, and bandgap factors. Finally, Section 5 summarizes the conclusions and prospects of the paper, highlighting the method’s potential for a wide range of nonlinear ultrasonic testing experiments within the 1~10 MHz frequency range.

## 2. Design Method of PC Filter

**Figure 2:**
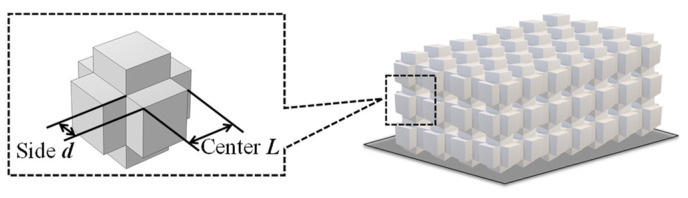
Geometric characteristics of cubic phononic crystals.

Based on the cubic phononic crystal proposed in Reference [40], the parameters of this configuration will be improved in this paper, and the main features will be extracted to summarize the band gap design method. Figure 2 illustrates a periodic structure consisting of a matrix (central mass block) and a scatterer (additional mass block), where the central matrix is represented as a cube. In this case, the geometric features are simplified as follows: the center length is represented by ‘L’ and the edge length is represented by ‘d’. It should be noted that the geometric size of the unit cell is in the range of 0.1 mm at MHz frequency for the phononic crystals based on Bragg scattering.

**Figure 3:**
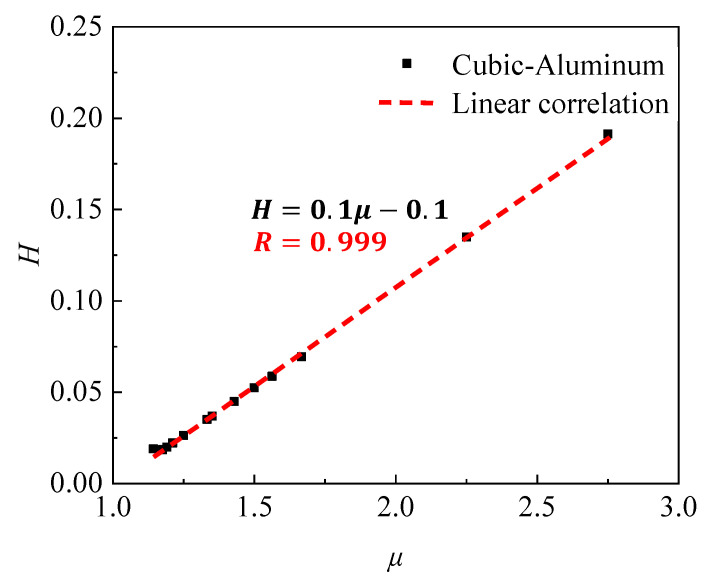
The linear correlation of dimensionless numbers.

Extensive numerical simulations and rigorous statistical investigations have revealed that PC filter designs exhibiting excellent filtering performance adhere to a consistent linear correlation characterized by dimensionless quantities. In the realm of PCs, which involve the coupled mechanisms of Bragg scattering, the interaction between the matrix mass block and the additional mass block is mutually influential. Notably, the center frequency of the bandgaps predominantly relies on the material properties and geometric characteristics of the phononic crystal. To address this, we introduce two dimensionless numbers for analysis. We define the dimensionless numbers *H* and *μ* as follows: $H=\left(L-d\right)\cdot f∕V$, μ=L∕d. Here, *L* is center length, *d* is side length, *f* is the bandgap’s center frequency, and *V* is the longitudinal wave velocity of the material. All measurements are expressed in the International System of Units with meters (m), hertz (Hz), and meters per second (m/s). Through an in-depth analysis of extensive datasets obtained from numerical investigations using a cubic-aluminum specimen, we establish an empirical formula:

(1) $$H=\alpha_{1}\mu^{\alpha_{2}}+\alpha_{3}$$

where $\alpha_{1}$, $\alpha_{2}$, and $\alpha_{3}$ are the coefficient of this formula. To ensure impedance matching, aluminum is selected as the material for designing PC filters. Figure 3 demonstrates the exceptional performance of PC filters with varying parameters, confirming their compliance with the relationship: H=0.1μ−0.1. Consequently, we establish a criterion that if the characteristics of a phononic crystal satisfy this dimensionless relationship, the filter performance is considered exceptional.

**Figure 4:**
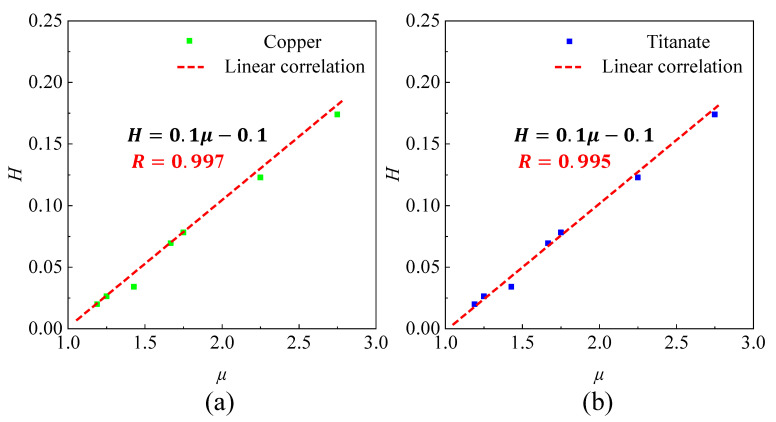
Linear correlation for copper (**a**) and titanium (**b**).

Subsequently, the feasibility of the dimensionless fitting curve is verified by considering the factors of material and bandgap type. In Figure 4a,b, the obtained fitting curves for different materials are found to be consistent with Figure 3 (where the material is aluminum). This observation provides evidence that the proposed dimensionless fitting curve is independent of the material selection.

**Figure 5:**
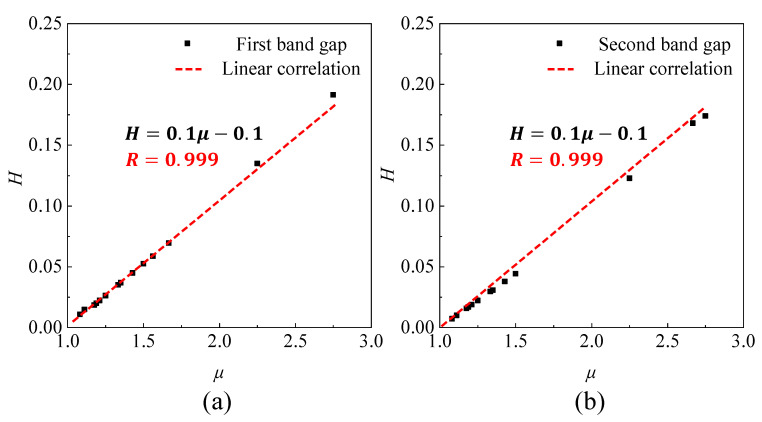
Linear correlation for the first band gap (**a**) and the second band gap (**b**).

Meanwhile, Figure 5a,b illustrate the dimensionless relationships among different bandgap types. The results demonstrate that for a given configuration, successful filtering can be achieved by designing the geometry to satisfy the fitting curve, regardless of the bandgap type. In summary, it can be concluded that the fitting relationship between *H* and *μ* remains consistent for the same configuration and is unaffected by changes in the material and bandgap type.

**Figure 6:**
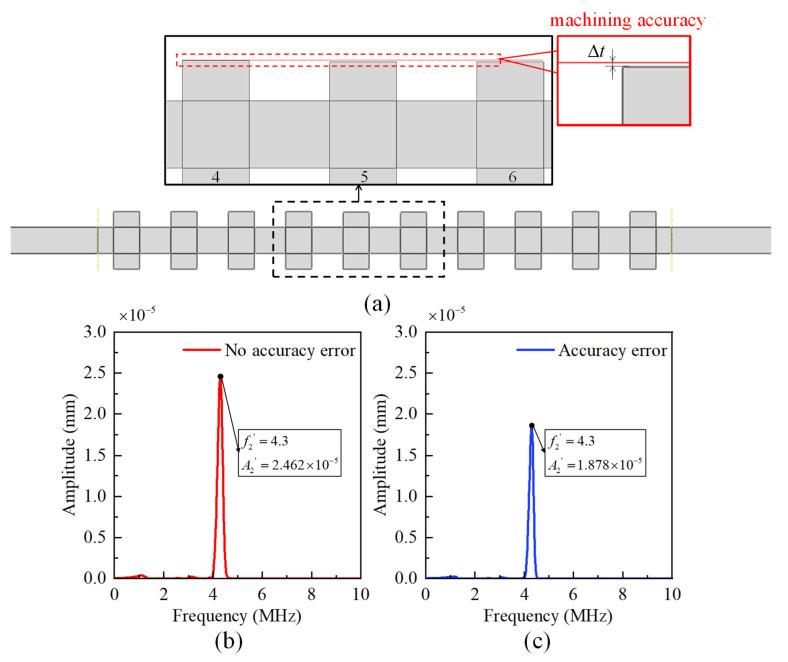
Influence of machining accuracy deviation on the filtering ability. (**a**) Structure with machining accuracy deviation; (**b**) Filtering results without machining accuracy deviation; (**c**) Filtering results with machining accuracy deviation.

Furthermore, geometric deviations in the phononic crystal filter caused by manufacturing-related mechanical errors could affect the central frequency, which is a crucial factor in the design process. In this work, the influence of machining accuracy deviation is examined, and the applicable conditions for this dimensionless relationship are supplemented. The employed fabrication technique is additive manufacturing (AM) at a sub-millimeter (0.1 mm) level, which is a higher precision 3D-printing method. In practical manufacturing, the primary limitation is machining accuracy. Figure 6a depicts a simulated structure with printing accuracy issues, where the phononic crystal at the fifth and sixth positions after the input signal exhibits a reduction in height in the upper part, which is smaller than the printing accuracy. The results as shown in Figure 6b,c indicate that machining accuracy errors have an impact on the preservation of the second harmonic, resulting in a 20% decrease in signal amplitude. However, this attenuation falls within an acceptable range. Therefore, existing 3D-printing technologies can be used for manufacturing, and estimates can be made for structural components smaller than 0.1 mm in design.

Due to the machining accuracy of AM additive manufacturing being within 0.1 mm in practical applications, and the deviation in machining accuracy not affecting the preservation of the second harmonic, it is feasible to estimate the geometric characteristics of the designed structure in engineering applications. This is reflected in the dimensionless fitting curve, where a certain deviation in material parameters from the fitting curve results in poor filtering performance of the filter. Therefore, the next step is to determine the range of estimation.

**Figure 7:**
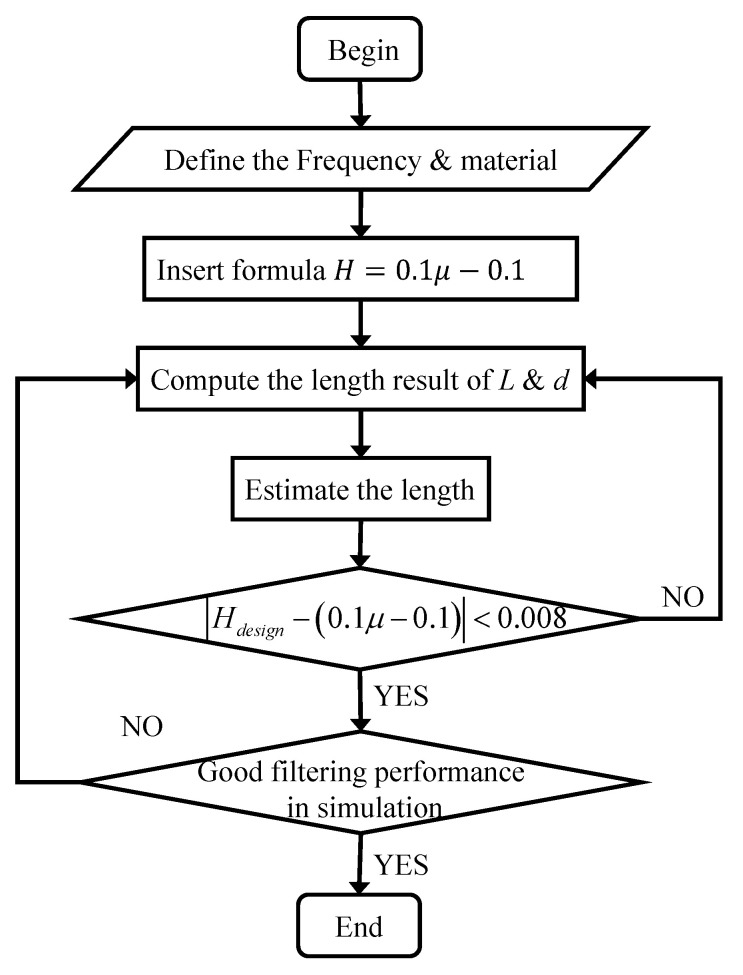
The flow chart of PC filter design.

By validating the statistical data, when $\left|H_{design}-\left(0.1\mu -0.1\right)\right|<0.008$, it is proved that the designed phononic crystal is effective within the allowable range of error. Among them, $H_{design}=\left[\left(L-d\right)\cdot f_{goal}\right]∕V$ and μ=L∕d, $f_{goal}$ is the frequency to be filtered out, and the dimensionless number $H_{design}$ is designed according to the dimensionless equation. Similarly, if the design of the PC filter meets the specified requirements, estimation can be performed with a decimal precision of 0.1 mm. Consequently, we summarize the entire filter design process here, as shown in Figure 7.

To sum up, different from the Reference [40], this paper investigates the factors to affect the cubic configuration. It is found that the band gap is related to the two dimensionless numbers. Then, according to the processing error of 3D printing, the allowable error is determined by the judgment criterion of phononic crystal design. Moreover, the ability of the ultrasonic filter can be verified by finite element simulations. Thus, two methods using finite element simulation will be established and employed in the next section.

## 3. Numerical Simulations

### 3.1. Bandgaps Properties Analysis

The phononic crystal, which is an important branch of metamaterials, has garnered significant attention for its ability to control the propagation of elastic waves in solids. In this section, our focus lies on the mechanism of PC filters, particularly leveraging the Bragg scattering mechanism. This study investigates the effects of material properties and geometric characteristics. A simple cubic lattice can be divided into the reciprocal lattice, the reduced Brillouin zone, and the irreducible Brillouin zone. It is important to note that the irreducible Brillouin zone is the smallest region into which the reciprocal lattice is symmetrically divided, which is capable of characterizing the characteristics of the entire lattice. To analyze the Bragg scattering mechanism in the irreducible Brillouin zone sweeping direction [42], we establish a three-dimensional model with Bloch boundary conditions using COMSOL Multiphysics™ 5.6. The modeling steps are as follows:

(a) Choose the built-in 3D Solid Mechanics module.
(b) Set material properties and boundary conditions. This 3D lattice possesses threefold symmetry in all directions and is made of aluminum. Floquet periodic boundary conditions ($\vec{u}’e^{-i\vec{k_{F}}\cdot \left(\vec{r}’-\vec{r}\right)}$) are applied in all three symmetric directions, corresponding to the characteristics of the Bragg boundaries, with a wave vector $\vec{k}=\left(\frac{\pi }{a},\frac{\pi }{a},\frac{\pi }{a}\right)$.
(c) Mesh the geometry. The minimum mesh size should be less than 1/10 of the wavelength.
(d) Conduct a frequency sweep analysis to obtain the characteristic frequency curve/band structure of the PC.

**Figure 8:**
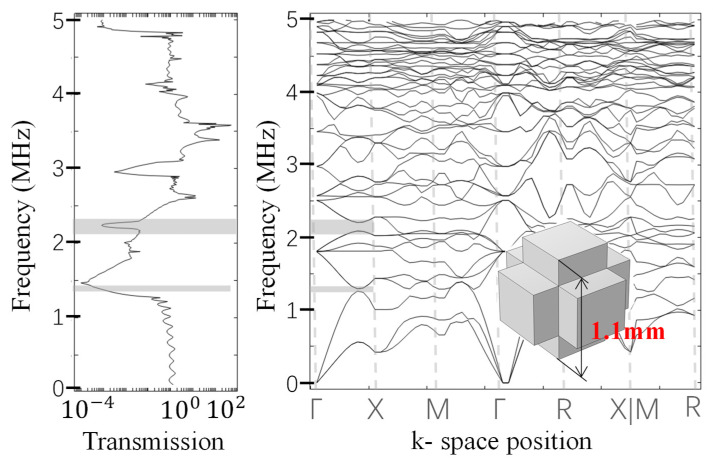
Bandgap characteristics of cubic lattice phononic crystal, left for signal transmission, right for dispersion curve.

Frequency analysis is performed to obtain the band diagram, depicted in Figure 8, where the gray areas represent the forbidden band regions. Signals within the forbidden band range cannot pass through the phononic crystal.

In this section, we use a high-performance phononic crystal structure as our model, constructed from aluminum with a central length (*L*) of 0.5 mm and a side length (*d*) of 0.3 mm.

### 3.2. Filtering Ability Verification

In this section, the time-domain finite element method is proposed as an alternative to actual experiments to demonstrate the filtering capability of the PC filter. We primarily define the filtering capability. Poor filtering capability indicates the presence of significant spurious signals or a significantly lower second harmonic compared with the spurious signals. Excellent filtering capability indicates negligible spurious signals and the maximum amplitude of the second harmonic signal.

**Figure 9:**
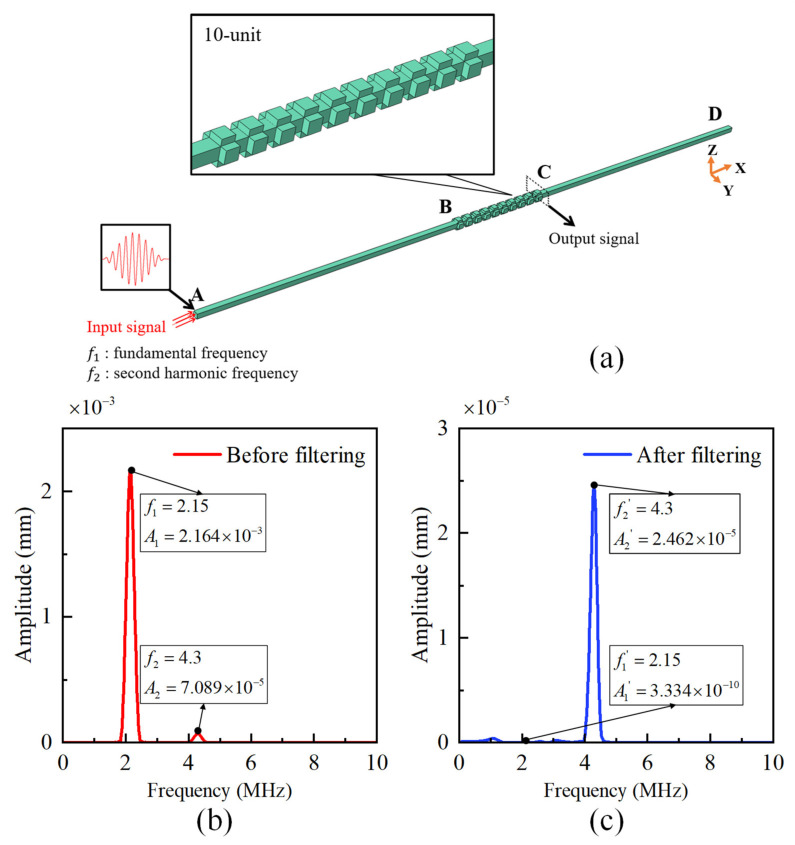
The time-domain finite element model verifies the filtering ability. (**a**) The prototype of PC filter. (**b**) Frequency domain diagram before filtering. (**c**) Frequency domain diagram of filtered signal.

The damage of early material degradation can be characterized by quadratic nonlinearity, which leads to higher harmonic frequencies, quasi-static frequencies, and mixing frequencies [43]. In other words, higher harmonic waves could be generated when the fundamental waves propagate in the damage region. Therefore, we establish the simulation model of the propagation process of nonlinear ultrasonic detection signals in the PC filter to assess its ability to filter out the fundamental frequency while preserving the second harmonic. Three-dimensional finite element analysis is conducted using the commercial finite element analysis software ABAQUS (Version 6.14, Dassault Systèmes Simulia Corp., Providence, RI, USA). Taking the cubic lattice filter as an example, Figure 9a represents the physical model of the propagation of ultrasonic detection signals in the PC filter. The model consists of 10 unit cells and propagation beams at both ends. The input signal is applied at point A and propagates through the transmission beam to reach the PC filter at point B. It passes through 10 unit cells and reaches point C, finally reaching the far end D through another transmission beam. The input signal at point A is a 20-period signal modulated by a Hanning window function, with a fundamental frequency of 2.15 MHz and a desired second harmonic frequency of 4.3 MHz, which can be obtained by numerical simulations of nonlinear ultrasonic wave [43]. The material chosen is aluminum (Al), and the wave velocity is set as *V* = 6300 m/s.

The computational results in Figure 9b,c demonstrate that the cubic lattice configuration of the PC filter effectively filters out the fundamental frequency of 2.15 MHz while preserving the second harmonic frequency of 4.3 MHz, indicating a strong filtering performance. Additionally, an analysis of the signal amplitude reveals the transmittance characteristics: for the fundamental frequency, it is completely reflected; for the second harmonic frequency, the transmittance reaches 35%, indicating the presence of reflection.

## 4. Parametric Studies

### 4.1. Mechanism Research

**Figure 10:**
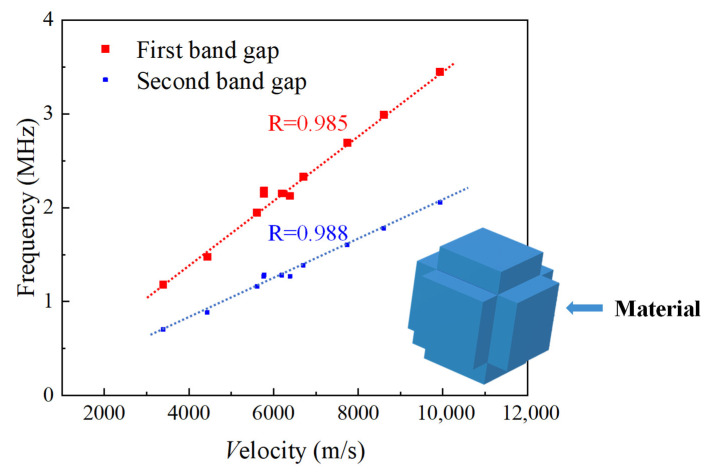
The correlation between material wave velocity and band gap center frequency.

In this section, we primarily focus on validating the proposed dimensionless design method. To begin, we explore the Bragg scattering mechanism within a frequency range of 1 MHz to 10 MHz, which guides our selection of a cell size on the order of 0.1 mm. Subsequently, we delve into the analysis of bandgap characteristics concerning different materials and geometric parameters. Here, the central frequency of the bandgap emerges as a crucial parameter of interest. We start by examining the influence of material properties on the bandgap, where the longitudinal wave velocity is determined by the formula:

(2) $$V={\left[E\left(1-\nu \right)∕\rho \left(1+\nu \right)\left(1-2\nu \right)\right]}^{1∕2}$$

where *E* represents the Young’s modulus, *υ* denotes the Poisson’s ratio, and *ρ* represents the material density. The validation results presented in Figure 10 reveal a highly correlated linear relationship between the longitudinal wave velocity *V* and the central frequency *f* of the bandgap. As the wave velocity increases, so does the central frequency of the bandgap, aligning with the dimensionless correlation we established earlier.

**Figure 11:**
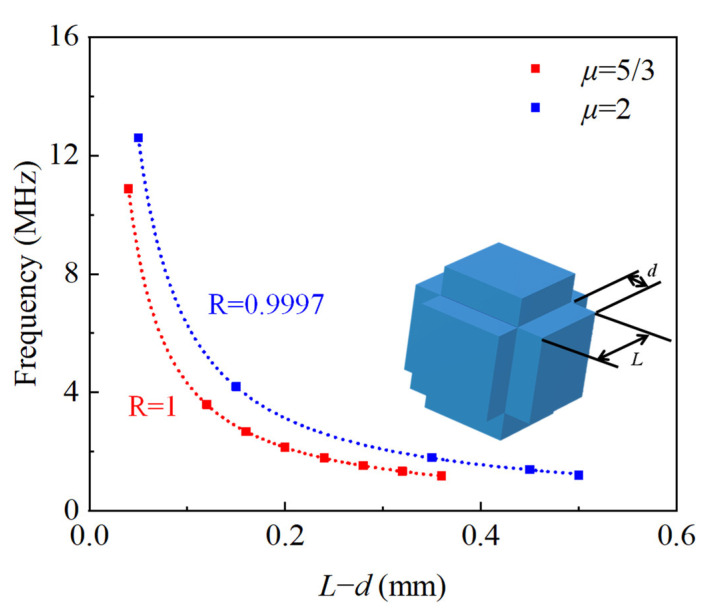
The correlation between geometry and band gap center frequency.

Next, we investigate the relationship between geometric parameters and the central frequency of the bandgap. As per our dimensionless correlation, when the value of (*L* − *d*) remains constant, there exists an inverse relationship between this value and the central frequency *f* of the bandgap. Figure 11 illustrates the validation results, confirming that the geometric parameter (*L* − *d*) indeed inversely affects the central frequency *f*. Hence, our findings validate the correctness of the geometric characteristics and the frequency matching of the dimensionless correlation.

### 4.2. Other Influencing Factors

**Figure 12:**
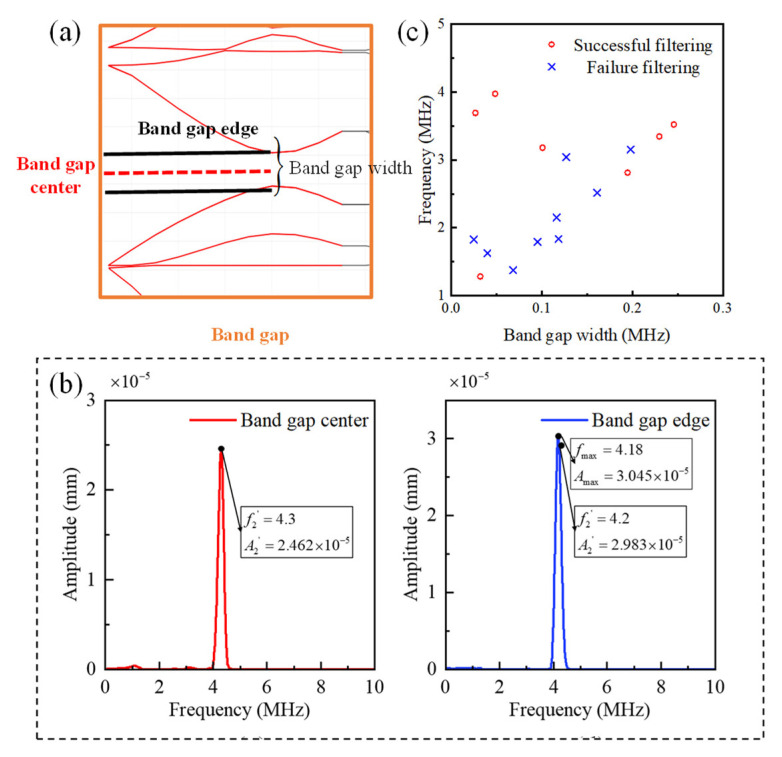
Influence of frequency. (**a**) Characteristics and corresponding names of band gaps. (**b**) The filtering result with band cap center and band cap edge. (**c**) The relationship between band gap width and filtering ability.

Furthermore, we conducted a thorough investigation into the impact of various factors on the filtering capability, including fundamental frequency selection, filter bandwidth, bandgap type, and machining precision errors. Our goal was to determine whether these factors significantly affect the design methodology of the ultrasonic filter. As shown in Figure 12a, the bandgap exhibits a distinct range of widths. Therefore, it is imperative to establish a methodology for selecting the bandgap frequency and assess how the bandgap width influences the filtering capability. Figure 12b provides clear evidence that filtering performance is optimized when the bandgap frequency is precisely positioned at the center of the bandgap. Consequently, selecting the bandgap center frequency as the key parameter for characterizing bandgap properties is a reasonable choice. Figure 12c illustrates the filtering capability under various bandgap widths, and the results unequivocally demonstrate that changes in the bandgap width have no discernible impact on filtering capability.

For bandgaps, there exist characteristics of the first bandgap, the second bandgap, and so on up to the nth bandgap. Table 2 presents the different bandgap types within the same structural design, as well as the filtering capabilities of the same bandgap type across different structural designs. The results indicate that the bandgap type is unrelated to the filtering capability. Hence, the linear relationship among dimensionless parameters is not influenced by the bandgap type as a contributing factor.

### 4.3. Cases for Design Methods

In this section, simulations were conducted using aluminum (AL) as the material to compare the filtering capabilities of phononic crystals that either satisfy or do not satisfy the dimensionless linear design method. The results of 13 sets of experiments are presented in Table 3. Each experiment targeted a specific frequency band to eliminate the influence of the frequency range. The last two columns in Table 3 indicate whether the structural design conforms to the dimensionless relationship and the corresponding filtering capability, demonstrating a significant correlation between them. Based on the above analysis, we can conclude that PC filters with a strong fitting correlation exhibit high filtering capability, and conversely.

Here, we present a successful example of phononic crystal design for nonlinear nondestructive testing. In this context, the typical fundamental frequency is 1 MHz, and the second harmonic frequency is 2 MHz. Employing our design method, we created a specific PC filter. The material chosen for this example had randomly assigned parameters, with the material properties: $E={150\times 10}^{9}$ Pa, ν=0.33, ρ=4940 kg/m^3^. By calculation, the longitudinal wave velocity is V=6707.4 m/s.

By substituting frequency f=1 MHz and velocity V=6707.4 m/s into linear correlation H=0.1μ−0.1, we could tentatively determine μ=5/3. From this, we obtained a center length L=1.1179 mm (approximately 1 mm), and a side length d=0.6707 mm (approximately 0.6 mm). We then inserted the results into error judgment criterion $\left|H_{design}-\left(0.1\mu -0.1\right)\right|=0.007<0.008$, which falls within the allowable error range.

**Figure 13:**
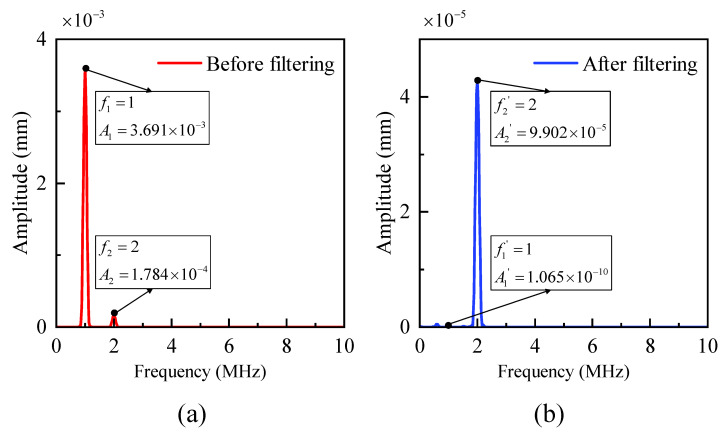
Frequency-amplitude. (**a**) Before filtering. (**b**) After filtering.

Subsequently, we validated the filtering capability through simulations using COMSOL (Version 5.6) and ABAQUS (Version 6.14) software. An analysis of the bandgap characteristics revealed that the frequency fell within the bandgap range, confirming that the geometric design met the required specifications. We then conducted a time-domain finite element analysis to verify the filtering capability, as depicted in Figure 13. In summary, this case serves as a successful demonstration that the designed filter, following the proposed design method, effectively filters out the 1 MHz fundamental frequency while preserving the 2 MHz second harmonic frequency.

## 5. Conclusions

In this study, based on a cubic phononic crystal, we established a model for the center frequency of bandgaps and proposed a design method for ultrasonic PC filters. Finite element simulations were conducted to validate the effectiveness of this design method in predicting bandgap center frequencies. The following conclusions can be drawn:

Firstly, inspired by the main mechanisms governing phononic crystal bandgap properties, namely Bragg scattering mechanisms of Bragg scattering, and through finite element analysis of parameters affecting these properties, we demonstrated that the interaction between the center mass block and additional mass block influences the bandgap center frequency. Therefore, considering parameters related to the material properties and geometric characteristics of PCs contributes to the establishment of dimensionless numbers for the bandgap center frequency.

Secondly, by utilizing PCs to filter out fundamental frequency signals within the bandgap frequency range while preserving second harmonic signals, we developed dimensionless number expressions and summarized a set of cubic phononic crystal design methods. Extensive finite element simulation results confirmed that the bandgap characteristics diagram verified the design conformity with the required bandgap center frequencies. Time-domain finite element simulations also exhibited the ultrasonic filtering characteristics of the phononic crystal. Hence, the PCs designed according to the proposed method entirely fulfilled the filtering criteria.

In summary, this study introduced dimensionless numbers related to the bandgap center frequency of ultrasonic cubic PCs and explored expressions between these dimensionless numbers through finite element simulations. Combining these findings with practical engineering needs, we established a filter design method. Based on the results of this research, we anticipate that the dimensionless design method presented here will provide theoretical and experimental simulation support for improving second harmonic modulation in the ultrasonic non-destructive testing of critical structural engineering components (aircraft and automobiles, etc.).

Moreover, we will focus on experimental validation of the proposed PC filter through the existing 3D-printing technology. And other factors, such as the size effect of PC filter, the impact of manufacturing errors on bandgap width, and the attenuation ability, will be explored and verified through experimental measurement. Design methods for other configurations will also be investigated. In the future, the proposed PC filter could achieve physical-control filtering capacity to separate fundamental wave and nonlinear wave signals, which is crucial for the damage monitoring of nonlinear ultrasonic systems.

## Figures and Tables

**Table 1:** Comparison of the filtering performance of this work with other research.

Reference	PC Configuration	Working Frequency	Parameter Analysis	Design Method
Lee et al. [38]	hierarchical PCs	20 kHz~10 MHz	-	-
Iglesias M. et al. [39]	cubic symmetry	0.6~7.5 MHz	-	-
Elizabeth J. et al. [40]	cubic, beam	100 kHz~5 MHz	-	-
Liu et al. [41]	plate	~3 kHz	geometry	deep learning method
This work	cubic	1~10 MHz	material/geometry	dimensionless method

**Table 2:** Filtering capability for various band gap types.

*L*/mm	*d*/mm	Band Gap Center Frequency/MHz	Band Gap Type	Filtering Ability
0.35	0.3	4.69	First	×
0.35	0.3	3.98	Second	×
0.35	0.3	1.83	Third	√
0.4	0.2	3.52	First	×
0.4	0.2	3.15	Second	√
0.45	0.2	3.69	First	×
0.45	0.2	3.34	Second	×
0.45	0.2	3.04	Third	√
0.5	0.25	2.81	First	×
0.5	0.25	2.52	Second	√
0.5	0.3	2.15	First	√
0.5	0.3	1.28	Second	×
0.5	0.4	1.63	First	√
0.5	0.4	1.37	Second	√

Note: The symbol “×” denotes poor filtering capability. The symbol “√” represents excellent filtering capability.

**Table 3:** Filtering capability of adapting linear correlation.

*L*/mm	*d*/mm	Fundamental Frequency/MHz	Second Harmonic Frequency/MHz	Adapt to Linear Correlation	Filtering Ability
0.4	0.2	3.5	7	No	×
0.45	0.2	3.34	6.68	No	×
0.45	0.2	3.69	7.38	No	×
0.5	0.3	1.28	2.56	No	×
0.6	0.2	2.97	5.94	No	×
0.45	0.2	3.34	6.68	No	×
0.4	0.2	3.15	6.3	Yes	√
0.45	0.3	1.82	3.64	Yes	√
0.5	0.3	2.15	4.3	Yes	√
0.5	0.4	1.54	3.08	Yes	√
0.5	0.4	1.62	3.24	Yes	√
0.5	0.4	1.37	2.74	Yes	√
1	0.6	1	2	Yes	√

Note: The symbol “×” denotes poor filtering capability. The symbol “√” represents excellent filtering capability.

## Data Availability

Data are contained within the article.
